# Description of the MIS EGS surgeon phenotype: how disruptive innovation can push the envelope of EGS practice

**DOI:** 10.1007/s00464-026-13174-w

**Published:** 2026-08-04

**Authors:** Neal Bartl, Lynnette Schiffern, William Sherrill, Hongmei Yang, Brent D. Matthews, Caroline E. Reinke

**Affiliations:** 1 Department of Surgery, Carolinas Medical Center, Charlotte, NC USA; 2 Department of Surgery, Wake Forest School of Medicine, Charlotte, NC USA; 3 Clinical Quality Analytics, Advocate Health, Charlotte, NC USA; 4 Department of Surgery, Wake Forest School of Medicine, 1025 Morehead Medical Plaza, Suite 300, Charlotte, NC 28203 USA

**Keywords:** Acute care surgery, Minimally invasive surgery, ACS systems, Surgical practice models

## Abstract

**Background:**

The structure of care for Emergency General Surgery (EGS) practices varies widely across hospital settings. EGS practices outside the setting of acute care surgery (ACS) services are less well understood. Within a multi-hospital setting, an innovative MIS-focused EGS practice was created to improve surgeon satisfaction, patient outcomes, and patient retention. We report successful implementation and expansion of an MIS EGS service across three hospital settings.

**Methods:**

Retrospective analysis of cases between January 2023 and December 2025 by a group of EGS surgeons across three hospitals using data available from the electronic medical record. Clinic volumes were identified for 2024/2025. Information on case volumes and types (elective vs. non-elective status, case type, OR block time) and clinic volumes and type of clinic visits were reported.

**Results:**

Over the 36-month period, 10,228 surgical cases were performed, the majority non-elective. A total of 8.4 FTE surgeons staffed three hospital EGS services, a total of four surgeons with clinical responsibilities Monday–Friday and two surgeons with clinical responsibilities on nights and weekends. Half-day clinics were staffed by surgeons for a total of 3.5 days per week across two sites with > 5000 clinic visits per year. 17.2% of elective cases had an ED visit within 8 weeks prior to their surgical intervention, with the most common diagnoses being biliary, gastrointestinal, and hernia related.

**Conclusion:**

Inclusion of elective cases within an EGS practice is feasible. Giving patients who present to the ED expedited follow-up and converting their surgical management to outpatient elective cases has many potential benefits. We believe this type of care model can allow for effective block utilization, efficient patient care, and surgeon satisfaction. Outlining the utility of a combination EGS + elective group practice for the hospital, patients, and surgeons can provide support for ongoing expansion of EGS focus outside of trauma settings.

The structure of care for Emergency General Surgery (EGS) practices varies widely across hospital settings. Incorporation of EGS care into teams already focused on other aspects of Acute Care Surgery (ACS)—trauma and critical care—is common [1, 2]. However, given the volume of non-trauma hospitals across the USA, many EGS patients are cared for at hospitals without dedicated ACS surgeons. Information regarding the implementation and structure of EGS practices outside of the trauma infrastructure are less well-described.

The efficacy of offloading tertiary center operative volume by transferring stable patients to community-based hospitals within a system has been previously described [3]. In our hospitals, many non-elective cases can undergo outpatient management—they are able to go directly from the ED to Preoperative Holding to OR to Recovery to home—without requiring any care in a hospital beyond that of the ED. The volume of EGS in the USA has been described using the National Inpatient Sample (NIS), notably omitting patients who undergo outpatient or observation status management, which may be more common at the lower-acuity hospitals that are facile in management of outpatient surgical care. In the era of increased use of minimally invasive surgery techniques for patients with EGS diagnoses [4–6] and remote triage of patients at free-standing emergency departments [7], understanding EGS care structures that support MIS access in community hospitals is important.

The goal of this study was to describe the creation and spread of a MIS-focused EGS surgical team across three hospital settings within a healthcare system. We aimed to outline the implementation and expansion of an MIS EGS service based on operative volumes, procedure type and elective status, clinic volumes, and staffing.

## Materials and Methods

The general surgery section consists of ten surgeons who cover emergency general surgery—interhospital transfers, emergency department (ED) consults, and inpatient consults—at two community hospitals and covers interhospital transfers, inpatient consults, and ED consults from Friday evening to Monday morning at the quaternary care flagship hospital (Table 1). The surgeons work across three hospitals allowing each surgeon time to work at the flagship hospital with a chief resident, a community hospital with a mid-level resident, and another community hospital where they operate with PAs or scrub techs. Each experience allows them to maintain a different skillset. The hospitals are close enough that patients seen in any clinic can be scheduled at any hospital allowing continuity. The general surgery section also covers clinic responsibilities for patients referred to and cared for at the three acute care hospitals and schedules elective general surgery cases from those clinics.

**Table 1:** General surgery coverage model across community and quaternary hospitals

	Weekdays	Nights and weekends
Hospital A 370 beds	2 surgeons 1 elective 1 emergency 2 APPs	1 surgeon 1 APP
Hospital B 185 beds	1 surgeon 1 APP 1 mid-level resident	0.5 surgeon 1 mid-level resident
Hospital C 800 + beds^a^	1 surgeon 1 APP 1 senior resident	0.5 surgeon 1 senior resident
MIS fellowship	5	< 5 years—3 surgeons 5–10 years—1 surgeon 20 + years—1 surgeon
Critical care fellowship	2	< 5 years—1 surgeon 10–20 years—1 surgeon
No fellowship	4	5–10 years—1 surgeon 10–20 years—1 surgeon 20 + years—2 surgeons

We performed a retrospective analysis of all operative cases performed by surgeons in this section between January 2023 and December 2025 using data available from the electronic medical record (EMR). Due to limitations of our case classification processes for inpatients and patients being transferred from a free-standing ED, operative cases were identified as elective vs. non-elective by the manner in which they were posted—cases that were associated with a “case request order” were considered non-elective as this indicates they were posted by someone on the inpatient service. Cases posted without such an order were considered elective as these would have been posted by our schedulers in clinic, which is completed by a fax or an email to central scheduling. Surgical procedures were identified by the case posting in the EMR and reflect the intended/anticipated procedures, and examination of final CPT codes was performed for cases that were posted as an exploratory laparotomy or diagnostic laparoscopy. Procedures were grouped into larger categories by manual review.

Clinic volumes were identified for 2024 and 2025. There are two clinics that serve the three acute care hospitals, and a clinic is available at one of the two locations 17.5 h per week, 13.5 of those hours with 1–2 attendings present per clinic (Table 1). The clinics see a broad range of general surgery conditions, including patients who presented to the ED initially and were safe for discharge with clinic follow-up—most commonly reducible hernias and biliary colic. An 8-week EMR “look-back” was performed for patients who had surgical intervention to determine the percent of patients who had an ED visit during that timeframe, the primary diagnosis of the ED visit, and the frequency of ED visits. ED visit primary diagnoses were similarly grouped into larger categories.

Information on case volumes, elective vs. non-elective status, case type, OR block time, block utilization, and clinic volumes were reported. No Institutional Review Board approval was required as the data utilized in this study does not meet human subject research.

## Results

**Fig. 1:**
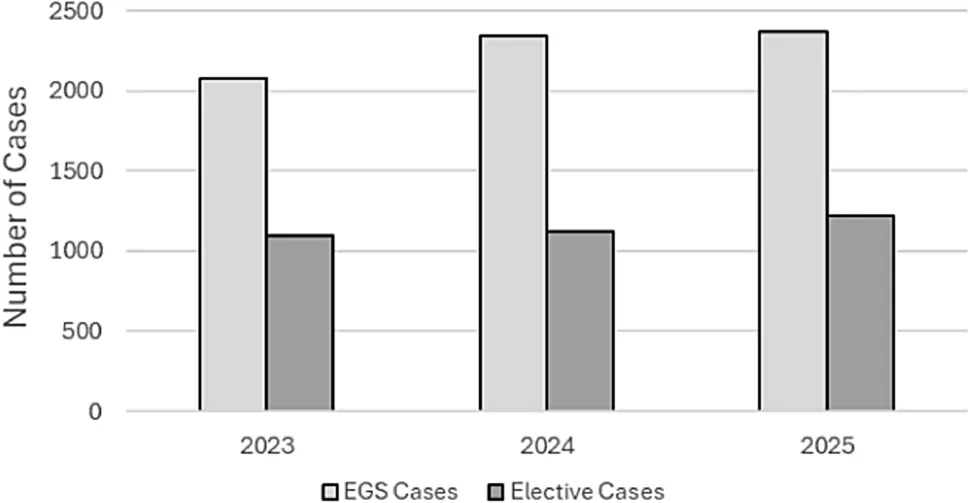
Number of EGS and elective cases by year (2023–2025). Notably, there was a substantial increase in EGS cases throughout the study duration. Elective case volume remained stable

A total of 8.4 FTE surgeons staffed 3 hospital EGS services, a total of 4 surgeons with clinical responsibilities Monday–Friday and 2 surgeons with clinical responsibilities on nights and weekends (Table 1). Over the 36-month period, 10,228 cases were performed in the operating rooms (Fig. 1), 34% elective and 66% non-elective (Table 2). Of emergent diagnostic laparoscopy and laparotomy cases, 25% were secondary to small bowel pathology, 16% due to colonic pathology, and 12% for adhesiolysis. The percent of operative practice that was elective ranged from 12 to 65% across individual surgeons. MIS approach for appendectomy and cholecystectomy was identified—99.6% of appendectomies and cholecystectomies were performed using a MIS approach, with 4.9% being robotic.

**Table 2:** Patient and disease characteristics of surgical cases

	EGS cases	Elective cases
	N = 6794 66.4%	N = 3434 33.6%
Age, mean/std	50.9 (19)	52.3 (17)
Male sex	46.6%	55.6%
ASA classification		
Healthy	7.6%	8.3%
Mild	40.6%	51.9%
Severe	35.5%	36.9%
Threatening/moribund	15.9%	2.6%
Number of comorbidities at surgery		
0	52.7%	47.7%
1	22.9%	28.1%
2	12.5%	14.9%
3	6.5%	6.0%
≥ 4	5.5%	3.3%
Specific comorbidities		
Diabetes	15.2%	12.6%
Hypertension	35.1%	38.4%
Coronary artery disease	8.8%	9.2%
Asthma	7.5%	11.2%
COPD	5.6%	4.2%
Chronic liver disease	5.7%	5.8%
Chronic kidney disease	8.2%	5.9%
Congestive heart failure	5.8%	2.9%
Primary procedure category^a^		
Appendectomy	1547 (22.8%)	98 (2.9%)
Cholecystectomy	2236 (32.9%)	876 (25.5%)
Colon procedure	144 (2.1%)	94 (2.7%)
Diagnostic laparoscopy	552 (8.1%)	49 (1.4%)
Exploratory laparotomy	909 (13.4%)	57 (1.7%)
Inguinal hernia repair	115 (1.7%)	858 (25%)
Skin/soft tissue	888 (13.1%)	610 (17.8%)
Umbilical hernia repair	49 (0.7%)	299 (8.7%)
Ventral hernia repair	64 (0.9%)	330 (9.6%)

^a^< 2% in both EGS and elective cases = diaphragmatic hernia, endoscopy, stomach procedures, enteral access, hiatal hernia, small bowel procedure, tracheostomy, and other

Half-day clinics were staffed by surgeons for a total of 3.5 days per week (plus 0.5 day APP clinic) across 2 sites with > 5000 clinic visits per year. Post-operative visits at our clinics ranged from 25.8 to 40.7% of the total patient population. Established patients made up 18.2–38.5%. Clinic visits at hospital C were more often post-operative due to a higher operative volume at that facility.

An ED visit 8 weeks prior to surgery was identified in 17.2% of elective cases, with the most common associated ED diagnoses being biliary, gastrointestinal, and hernia related (in total accounting for 72.1% of ED visits).

## Discussion

Optimal models of care for EGS have yet to be elucidated, and likely there are multiple options that can successfully deliver excellent patient care while also offering professional satisfaction for surgeons and effective care delivery within a hospital or healthcare system. Data to date have suggested that a team dedicated to care of the acute patient can be associated with improved outcomes, though the underlying mechanism for this finding is not yet understood [8]. This study described the clinical care structure of a group of MIS-focused surgeons providing primarily EGS care, while incorporating clinic and strategic elective surgical care into the practice infrastructure.

In our practice, > 10,000 cases were performed over a three-year period, with a mix of elective and emergency surgical procedures. This mix allowed surgeons to maintain high case volume across a wide range of operative procedures. While the rate of MIS approach for each procedure was beyond the scope of this study, the MIS approach for appendectomy and cholecystectomy were consistent with the literature, approaching 100%. Use of MIS in EGS is increasing across many diagnoses [4, 5], and concerns about risks of pneumoperitoneum are primary limited to the patients with hemodynamic instability. Surgeon surveys have suggested that training in laparoscopy for emergency surgeons would be valuable [9] and that the ability to have an elective case volume would make an EGS job more attractive [10]. The combined elective and emergency surgery model additionally allows for rapid access to elective surgical care for patients discharged from the ED with surgical diagnoses.

Longstanding evidence demonstrates the importance of the volume–outcome relationship within surgical practice and our model capitalizes on this, incorporating elective counterparts as part of the routine surgical case volume. Elective surgical practice may benefit from a focused area of expertise but can also “silo” surgeons into performing a narrow case mix, limiting experience with the EGS patient. EGS cases provide a platform for broad utilization of surgical skills as well as maximizing operative block-time utilization. Expansion of EGS structures to both community hospitals were initially hampered by limited access to OR time but were quickly remedied as our utilization metrics demonstrated full use of allocated OR time. Increasing OR availability both for EGS and elective cases (especially those posted in an accelerated fashion) likely leads to increased patient satisfaction.

While our data does not have detail on patient diagnoses, oncologic pathology occasionally presents as a surgical emergency, and management decisions can be complex, necessitating the weighing of immediate treatment options with longer-term management and outcomes. In this clinical setting, we address these challenges with collaborative pathways created with surgical oncology for specific diagnoses (colorectal cancer and esophageal/gastric cancers), availability of surgical oncology colleagues for consultation regarding decision-making and occasionally technical assistance, and routine monitoring of oncologic process metrics (margins and nodal harvest) as part of our oncologic quality registry.

The surgical clinic is the backbone for OR coordination and scheduling within our practice. We see a broad mix of referrals for elective cases as well as emergency department follow-up visits. These patients are triaged based on acuity and can often be seen within one to two weeks of ED presentation. The most common diagnoses seen in the ED then scheduled for short-term operative intervention are biliary disease and ventral hernias. The practice of elective follow-up, but with a short timeframe, allows for pre-operative risk stratification and operative planning for elective intervention while minimizing the volume of return visits to the ED. In order to see the large volume of clinic patients, referrals and follow-ups, our team utilizes a clinical care model in which 2–3 surgeons are scheduled to be present during each clinic. This has allowed us to double- and triple-book clinic appointments while still running clinic on time. This also provides flexibility for one surgeon to leave if any emergencies or intra-operative consults occur during the clinic.

The goal of this work was to describe an EGS practice which incorporates an elective practice and has a preponderance of MIS Fellowship-trained surgeons. It was not designed or powered to assess the impact of surgical team structure and training on rates of MIS approaches or patient outcomes, which is a limitation of the evaluation of the “success” of such a structure.

We believe that minimally invasive surgeons are well suited for care of EGS patients. Elective practice provides the foundational technical skills to manage the broad variety of pathology seen within the EGS patient population. In turn, the volume of elective and emergency cases further expand the surgeon’s knowledge base and hone technical skill. This may be especially important in hernias and colon procedures, where both MIS and open techniques are commonly used. In an era when surgical practice is often hyper-specialized, our hybrid model provides an avenue to operate at the optimal potential of a general surgeon while still having local expertise outside of our group for sub-specialty general surgical care. With support of the health system, hospital, and department an innovative group of surgeons created a MIS EGS niche clinical practice combining elective and EGS care, and future studies will be needed to determine the impact on surgeon satisfaction, patient outcomes, and patient retention. Understanding EGS care structures across a variety of settings, and their association with surgeon and patient outcomes, is important.

## Conclusion

Inclusion of elective cases within an EGS practice is feasible. Giving patients who present to the ED expedited follow-up for non-emergent conditions and converting their surgical management to outpatient elective cases has many potential benefits and is an important component of population health. Conversely, offering elective cases within an EGS-focused practice allows for a broad case mix and opportunities to utilize MIS skills in both settings. Clearly outlining the benefits of a combination EGS + elective group practice for the hospital, patients, and surgeons can provide support for ongoing expansion of EGS focus outside of trauma settings.
